# COVID-19 Vehicle Based on an Efficient Mutual Authentication Scheme for 5G-Enabled Vehicular Fog Computing

**DOI:** 10.3390/ijerph192315618

**Published:** 2022-11-24

**Authors:** Mahmood A. Al-Shareeda, Selvakumar Manickam

**Affiliations:** National Advanced IPv6 Centre (NAv6), Universiti Sains Malaysia, George Town 11800, Penang, Malaysia

**Keywords:** COVID-19 vehicle, fog server, 5G-enabled vehicular network, authentication

## Abstract

The COVID-19 pandemic is currently having disastrous effects on every part of human life everywhere in the world. There have been terrible losses for the entire human race in all nations and areas. It is crucial to take good precautions and prevent COVID-19 because of its high infectiousness and fatality rate. One of the key spreading routes has been identified to be transportation systems. Therefore, improving infection tracking and healthcare monitoring for high-mobility transportation systems is impractical for pandemic control. In order to enhance driving enjoyment and road safety, 5G-enabled vehicular fog computing may gather and interpret pertinent vehicle data, which open the door to non-contact autonomous healthcare monitoring. Due to the urgent need to contain the automotive pandemic, this paper proposes a COVID-19 vehicle based on an efficient mutual authentication scheme for 5G-enabled vehicular fog computing. The proposed scheme consists of two different aspects of the special flag, SF = 0 and SF = 1, denoting normal and COVID-19 vehicles, respectively. The proposed scheme satisfies privacy and security requirements as well as achieves COVID-19 and healthcare solutions. Finally, the performance evaluation section shows that the proposed scheme is more efficient in terms of communication and computation costs as compared to most recent related works.

## 1. Introduction

A major worldwide health crisis unlike any other in history is currently affecting everyone on Earth, causing chaos in people’s lives and spreading agony. The World Health Organization (WHO) has described the coronavirus disease COVID-19 as constituting fatal and highly contagious pandemic [1,2,3,4]. It has been responsible for thousands of deaths worldwide and is still wreaking havoc on communities [5,6,7]. The majority of countries have to date deployed tight detection and surveillance methods for public spaces such as airports and train stations, as well as means of public transit including airplanes, subways, and trains [8,9,10].

The special benefits of the fifth-generation 5G-enabled vehicular network could be taken into consideration with the pressing needs for pandemic control [11,12,13,14]. That is, the two key components of COVID-19-control—advanced healthcare monitoring and infection tracking—could be carried out by a 5G-enabled vehicular network without requiring direct human contact. As a result, the working staff is spared the perilous task of conducting surveillance at each inspection station. Instead, if required, each instance of fog computing might be given the role of the automatic checkpoint. In densely populated metropolitan cities and regions, the 5G-enabled vehicular network is regarded as a crucial element of the future vehicular ad hoc network (VANET) in order to deliver innovative vehicular data processing and traffic management analyses [15]. A major vehicular network infrastructure is composed of a 5G-base station, fog server, trusted authority (TA), and terminal vehicle equipped with the onboard unit (OBU) [16,17]. Vehicular networks provide for two different types of data transfer: vehicle-to-vehicle and vehicle-to-fog server through a 5G base station.

In order to address security challenges in the vehicular network, numerous studies using various safety procedures and cryptographic algorithms have recently been conducted. However, the criteria required for the 5G-enabled vehicular network’s actual implementation have not yet been fully taken into account. Vehicular networks’ prospective applications and useful uses should be investigated in light of the COVID-19 pandemic’s urgent global predicament. Measurements for vehicular networks and their related extensions should be developed now that the transportation system has emerged as one of the most hazardous scenarios for viral surveillance and infection tracking.

Due to the urgent need to contain the automotive pandemic, this paper will propose a COVID-19 vehicle-based efficient mutual authentication scheme for 5G-enabled vehicular fog computing. The proposed scheme consists of two different aspects according to a special flag (SF) value that involves a mutual authentication process. Based on the SF value, we consider the type of vehicle with SF = 0 and SF = 1 as the normal and COVID-19 vehicles, respectively. The main contributions of this paper are listed as follows:

- COVID-19 vehicle-based efficient mutual authentication scheme is proposed for 5G-enabled vehicular fog computing.
- A scheme does not only satisfy privacy and security requirements but also achieves COVID-19 and healthcare solutions.
- Performance evaluation section shows that the proposed scheme is more efficient in terms of communication and computation costs as compared to most recent related works.

The remainder of this paper is organized as follows: In Section 2, we review some mutual authentication schemes for vehicular networks. Section 3 describes the system model and mathematical methods of our proposal. Section 4 proposes a COVID-19 vehicle-based scheme for 5G-enabled vehicular fog computing. Section 5 evaluates security analysis and the comparison of proposal. Section 6 analyses the communication and computation overhead. Lastly, we provide the conclusion of this paper in Section 7.

## 2. Related Work

In this section, we review some mutual authentication schemes for vehicular networks as follows. Wang et al. [18] designed a mutual authentication system based on local identity by assigning unique long-term certification from TA to RSU and a vehicle in the enrolment process. Ming et al. [19] designed certificateless cryptography to provide communication security in a vehicular network. This scheme can simultaneously verify a large number of received messages by RSU. Al-Shareeda et al. [20] designed a privacy-preserving communication scheme by injecting fake messages to achieve unobservability requirements. This scheme has massive overhead in terms of communication and computation costs. For V2V communication, Ali and Li [21] proposed a signature based on an identity scheme using a secure hash function in high-density areas with high traffic. Zhang et al. [22] constructed a fuzzy logic mathematical approach to share security data among vehicles in a group based on a 5G-enabled model. Li et al. [23] presented a provable authentication scheme to provide both the security and privacy required for vehicular networks. Cui et al. [24] designed a content-sharing scheme for reliable communications in 5G-enabled vehicular networks. This scheme picked sophisticated proxy vehicles and requested them for content services. Alshudukhi et al. [25] constructed combined schemes between RSU-based and OBU-based schemes by exchanging temporary keys to sign messages and verify signatures during a short period. Al-Shareeda et al. [26] proposed a data-sharing scheme to secure 5G-enabled vehicular networks without using roadside units (RSUs). This scheme preloaded a large number of pseudonym IDs and relevant private keys to the registered vehicle during the key generation phase. However, this scheme has a massive overhead in verifying the messages exchanged among vehicles in terms of single verification and batch verification.

However, these mutual authentication schemes have massive performance overheads in terms of communication and computation costs. Additionally, none of these schemes address the COVID-19 virus and healthcare solutions for vehicular networks to exchange messages with trusted parts. In order to cope with these issues, this paper will propose efficient mutual authentication to sign messages and verify the signature. Additionally, our proposal consists of two different aspects according to the special flag (SF) value that involves a mutual authentication process. Based on the SF value, we consider the type of vehicle to be a normal vehicle when SF = 0 and a COVID-19 vehicle when SF = 1.

## 3. Background

This section introduces the system model and mathematical method used in the proposed scheme as follows:

### 3.1. System Model

**Figure 1:**
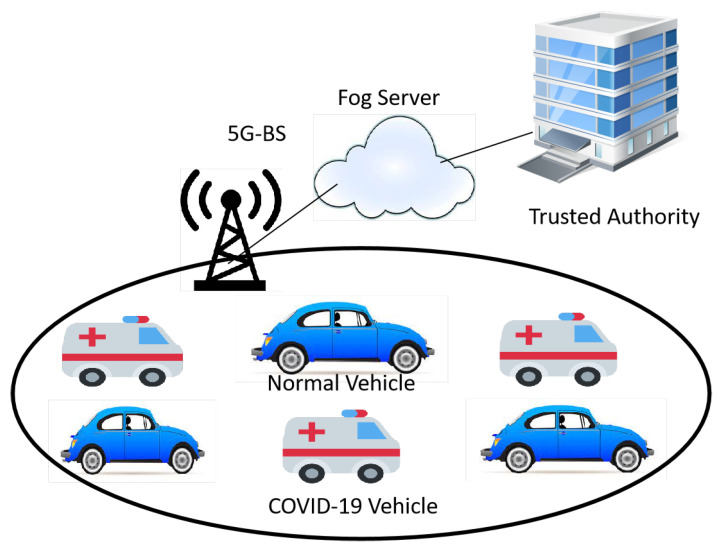
System Model of Proposed Scheme.

As shown in Figure 1, the system model of the proposed scheme consists of four main entities, namely the trusted authority (TA), fog server, 5G-base station (5G-BS), and onboard unit (OBU) that are equipped in each vehicle. The distribution of these entities is as follows.

- Trusted Authority (TA): It is the sole authority that is a trustworthy third party and can decipher an OBU’s identity from encryption. It is in charge of producing system parameters and has significant computing and storage capabilities.
- Fog Server: The fog server is regarded as a completely reliable entity that will assist TA in disclosing the names of the signers in our endeavour. In order to generate the pseudonym IDs of the vehicles over mutual authentication via 5G-BS, the fog server has its master key preloaded by TA. Our work relies on the public key of the fog server for the verification process.
- 5G-Base Station (5G-BS): The 5G-BS is a reliable infrastructure that has been placed beside roads. Without any storage or computing, it functions as a bridge between entities.
- Onboard Unit (OBU): OBUs are installed in every car; they are secure and cannot be removed or interfered with. OBUs are wireless logical units that use the DRSC and 5G protocols to communicate with other OBUs and fog servers via 5G-BS, respectively. In this paper, there are two types of vehicles called normal vehicles and COVID-19 vehicles. In this paper, the terms of CVPS and NVPS will be used to refer to COVID-19 vehicles and normal vehicles, receptively.

### 3.2. Mathematical Methods Used

In this section, we describe the mathematical methods used in the proposed scheme as follows.

#### 3.2.1. Elliptic Curve Cryptography (ECC)

Elliptic curve cryptography (ECC) was first conceived in 1958 by Miller [27]. Designing security procedures and digital signatures now frequently uses this kind of cryptographic technology. For more details about ECC, we recommend reading [20,28,29].

#### 3.2.2. Hash Cryptographic Function

One of a number of hash functions created by the US national security agency (NSA), Secured Hash Function 512, is a component of the US Federal Information Processing Standard [30,31,32].

## 4. Proposed Scheme

**Figure 2:**
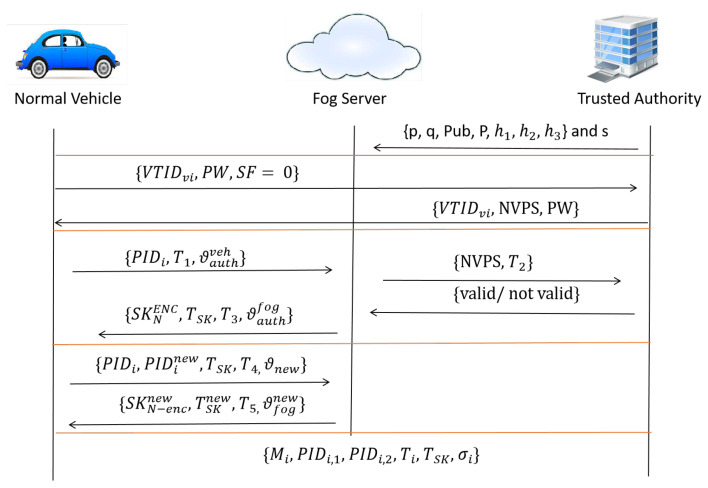
Normal Vehicle Process.

**Figure 3:**
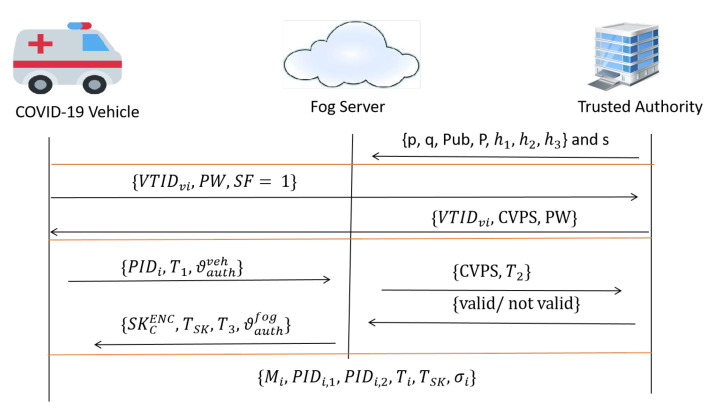
COVID-19 Vehicle Process.

In this section, we describe our proposed COVID-19 vehicle based on an efficient mutual authentication scheme for 5G-enabled vehicular fog computing in detail. In this paper, seven phases are included in our proposal, namely setup, enrolment, mutual authentication, updating private key, message signing, signature verification, and identity revocation phases. The seven phases in our proposal consist of two different aspects according to the special flag (SF) value that involves the mutual authentication process. Based on the SF value, we consider the type of vehicle to be a normal vehicle when SF = 0 and a COVID-19 vehicle when SF = 1.In the setup phase, the two types of SF values (e.g., 0 and 1) are used in this phase, in which TA is in charge of creating system parameters and broadcasting to all fog servers and vehicles. Furthermore, the two types of SF values are used in the setup phase. In the enrolment phase, TA is in charge of registering participating vehicles by applying both SF values to output two types of pseudonym IDs. Once the vehicle wishes to authenticate itself with the system, the vehicle will send its security parameters (e.g., password, public key, and SF value) to a nearby fog server through the wide communication range of 5G-BS. Based on the SF value, the vehicle then obtains a pseudonym ID to conceal its true identity. In the mutual authentication phase, the enroled vehicle will request the private key that is also based on the SF value. If the SF value equals 0, approval is granted for both fog servers and vehicles to broadcast messages, while if SF value equals 1, approval is allowed for only fog servers. In the message signing phase, the vehicle will start to create and sign a message to generate a signature tuple. Therefore, the verifier checks the validity and authenticity of the message based on the signature in the signature verification phase. In updating the private key phase, once the timestamp of the private key is close to being expired, the normal vehicle requests that its private key is renewed, while the COVID-19 vehicle skips this phase, which as a result, enhances performance and satisfies the security and privacy properties for all COVID-19 vehicles. Finally, in the identity revocation phase. Our proposal has the ability to trace and revoke any attacker (e.g., malicious normal or COVID-19 vehicles) that appears hostile to or pierced by the system. More precisely, our proposal expels and prevents the malicious vehicle from updating its private key in the previous phase. Figure 2 and Figure 3 show normal vehicle and COVID-19 vehicle processes, respectively. These phases can be described in more detail as follows:

### 4.1. Setup Phase

The TA executes this phase to create the public parameters of the system. To maintain the security of the system in 5G-enabled vehicular fog computing, TA always frequently updates system parameters. The process of this phase is as follows:

- TA defines the equation of the elliptic curve EC $y^{2}=x^{3}+ax+b$mod*p*, where $a,b\in Z_{q}^{*}$.
- TA chooses the pairs of large primary numbers (e.g., *p* and *q*) based on an additive group *G*.
- TA selects a random number *s* as the system’s private key and computes the concerned system’s public key Pub=s·P.
- TA picks three hash cryptographic functions $\left(e.g.,h_{1},h_{2},h_{3}\right)$ as $h_{1}:G\to Z_{q}^{*}$$h_{2}:{\left\{0,1\right\}}^{*}\times {\left\{0,1\right\}}^{*}\times G\to Z_{q}^{*}$$h_{3}:{\left\{0,1\right\}}^{*}\to Z_{q}^{*}$.
- TA securely saves the system’s private key *s* to all fog servers.
- Finally, TA broadcasts the system’s parameters (*p*, *q*, Pub, *P*, $h_{1}$, $h_{2}$, $h_{3}$) to all fog servers through wire communication.

### 4.2. Enrolment Phase

Any new user who wishes to join 5G-enabled vehicular fog computing must first complete a number of legality-checking tasks before registering. This process is as follows:

- The user submits a joining message including the vehicle’s true identity $VTID_{v_{i}}$, password (e.g., PW), and an SF value to TA through a secured channel. Where values of SF = 0 and SF = 1 indicate the normal vehicle and COVID-19 vehicle, respectively.
- In the case that a value of SF = 0, TA first verifies the vehicle’s true identity $VTID_{v_{i}}$ and then computes the normal vehicle’s pseudonym ID $NVPS=h_{1}(VTID_{v_{i}}\left||s\right)$. Finally, TA preloads personal information $\left(VTID_{v_{i}},NVPS,PW\right)$ to the TPD of OBU for a normal registered vehicle.
- In the case of a value of SF = 1, TA first verifies the vehicle’s true identity $VTID_{v_{i}}$ and then computes the COVID-19 vehicle’s pseudonym ID $CVPS=h_{1}(VTID_{v_{i}}\left||s\right)$. Finally, TA preloads the personal information $\left(VTID_{v_{i}},CVPS,PW\right)$ to the TPD of OBU for the COVID-19 registered vehicle.
- Finally, TA saves the sensitive data $\left(VTID_{v_{i}},NVPS,PW\right)$ and $\left(VTID_{v_{i}},CVPS,PW\right)$ into a normal vehicle registration list and COVID-19 vehicle registration list, respectively.

### 4.3. Mutual Authentication Phase

Once vehicle $v_{i}$ wishes to authenticate itself with the system, the following process should be executed:

- Vehicle $v_{i}$ picks random number $r\in Z_{q}^{*}$ and calculates the two pseudonym IDs $PID_{i}$ ($PID_{i,1}$ and $PID_{i,2}$ (as Equations (1) and (2), respectively) based on the SF value to the normal vehicle and COVID-19 vehicle, respectively) in order to conceal the vehicle’s true identity.
  (1) $$\begin{array}{cc} PID_{i} & =(PID_{i,1},PID_{i,2})\\ \; & =(r\cdot P,NVPS⊕h_{1}\left(r\cdot Pub\right)) \end{array}$$
  (2) $$\begin{array}{cc} PID_{i} & =(PID_{i,1},PID_{i,2})\\ \; & =(r\cdot P,CVPS⊕h_{1}\left(r\cdot Pub\right)) \end{array}$$
- Vehicle $v_{i}$ generates signature $\delta_{auth}^{veh}$ as Equations (3) and (4) according to the normal or COVID-19 vehicle, respectively.
  (3) $$\delta_{auth}^{veh}=h_{2}(NVPS||PID_{i}\left|\right|T_{1})$$
  (4) $$\delta_{auth}^{veh}=h_{2}(CVPS||PID_{i}\left|\right|T_{1})$$
- Vehicle $v_{i}$ transmits $\left(PID_{i},T_{1},\delta_{auth}^{veh}\right)$ to the nearest fog server $FS_{j}$ through the wide-range communication of 5G-BS.
- Once the fog server $FS_{j}$ receives $\left(PID_{i},T_{1},\delta_{auth}^{veh}\right)$ from the vehicle $v_{i}$, the $FS_{j}$ first checks the freshness of the timestamp as Equation (5). Where $T_{▽}$ is the predefined delay time and $T_{r}$ is the received time of $\left(PID_{i},T_{1},\delta_{auth}^{veh}\right)$,
  (5) $$T_{i}>T_{r}-T_{▽}$$
- Once $T_{1}$ is valid, the fog server $FS_{j}$ computes $NVPS=PID_{i,2}⊕h_{1}\left(sPID_{i,1}\right)$ or $CVPS=PID_{i,2}⊕h_{1}\left(sPID_{i,1}\right)$ to check the validity of signatures as Equation (6) or Equation (7).
  (6) $$\delta_{auth}^{veh}\overset{?}{=}h_{2}(NVPS||PID_{i}\left|\right|T_{1})$$
  (7) $$\delta_{auth}^{veh}\overset{?}{=}h_{2}(CVPS||PID_{i}\left|\right|T_{1})$$
- If the above equation is false, the fog server $FS_{j}$ discards the message; otherwise, it sends $\left(NVPS,T_{2}\right)$ or $\left(CVPS,T_{2}\right)$ to TA based on the value of SF.
- Once TA receives the security parameters from fog server $FS_{j}$, TA checks the newness of timestamp $T_{2}$ and then verifies the match stored values NVPS or CVPS into the normal vehicle registration list and COVID-19 vehicle registration list, respectively.
- TA sends valid or not valid to fog server $FS_{j}$ according to the above verification.
- If the message is verified, fog server $FS_{j}$ will generate the private key $SK_{N}$ (as Equation (8)) for the normal vehicle that has SF = 0 or $SK_{C}$ (as Equation (9)) for the COVID-19 vehicle that has SF = 1, where $T_{SK}$ is a lifetime of the private key.
  (8) $$SK_{N}=s\cdot h_{2}(PID_{i,1}||PID_{i,2}\left|\right|T_{SK})$$
  (9) $$SK_{C}=s\cdot h_{2}(PID_{i,1}||PID_{i,2})$$
- The fog server $FS_{j}$ encrypts the private key as Equation (10) or Equation (11) based on the type of vehicle.
  (10) $$SK_{N}^{Enc}=SK_{N}⊕h_{2}\left(s\cdot PID_{i,1}\right)$$
  (11) $$SK_{C}^{Enc}=SK_{C}⊕h_{2}\left(s\cdot PID_{i,1}\right)$$
- The fog server $FS_{j}$ sends $\left(SK_{N}^{Enc},T_{SK},T_{3},\delta_{auth}^{fog}\right)$ and $\left(SK_{C}^{Enc},T_{3},\delta_{auth}^{fog}\right)$ to the normal vehicle and COVID-19 vehicle, receptively, where $\delta_{auth}^{fog}$ is calculated as Equations (12) and (13).
  (12) $$\delta_{auth}^{fog}=h_{2}(SK_{N}\left|\right|T_{3}\left|\right|T_{SK})$$
  (13) $$\delta_{auth}^{fog}=h_{2}(SK_{C}\left|\right|T_{3})$$
- In case of the normal vehicle (e.g., SF = 0), the vehicle $v_{i}$ decrypts the private key $SK_{N}=SK_{N}^{Enc}⊕h_{2}\left(r\cdot Pub\right)$ and checks the signature $\delta_{auth}^{fog}\overset{?}{=}h_{2}(SK_{N}\left|\right|T_{3}\left|\right|T_{SK})$.
- In the case of the COVID-19 vehicle (e.g., SF = 1), the vehicle $v_{i}$ decrypts the private key $SK_{C}=SK_{C}^{Enc}⊕h_{2}\left(r\cdot Pub\right)$ and checks signature $\delta_{auth}^{fog}\overset{?}{=}h_{2}(SK_{C}\left|\right|T_{3}\left|\right|T_{SK})$.

Note that the normal vehicle that has an SF = 0 will exchange data among the vehicles using its private key $SK_{N}$, while not required to broadcast messages (e.g., the velocity, location, speed, direction) to others for COVID-19 vehicles. Thus, the COVID-19 vehicle only sends a message (e.g., healthcare information) to the nearest fog server through the communication range of 5G-BS using its private key $SK_{C}$.

### 4.4. Updating Private Key Phase

In this section, when lifetime $T_{SK}$ is close to expiring, the normal vehicle only executes this phase to update its private key $SK_{N}$, while the COVID-19 vehicle skips this phase. The normal vehicle will update the private key with the nearest fog server through wide-range communication by 5G-BS without being required to touch TA. This process is as follows:

- Vehicle $v_{i}$ selects random number $r_{new}\in Z_{q}^{*}$ and calculates the two pseudonym IDs $PID_{i}^{new}$ ($PID_{i,1}^{new}$ and $PID_{i,2}^{new}$) with Equation (14) in order to conceal the vehicle’s true identity.
  (14) $$\begin{array}{cc} PID_{i}^{new} & =(PID_{i,1}^{new},PID_{i,2}^{new})\\ \; & =(r_{new}\cdot P,NVPS⊕h_{1}\left(r_{new}\cdot Pub\right)) \end{array}$$
- Vehicle $v_{i}$ sends $\left(PID_{i},PID_{i}^{new},T_{SK},T_{4},\delta_{new}\right)$ to nearest the fog server $FS_{j}$, where $\delta_{new}$ is calculated as Equation (15).
  (15) $$\delta_{new}=SK_{N}+r\cdot h_{2}(PID_{i,1}^{new}||PID_{i,2}^{new}\left|\right|T_{4})$$
- Once receiving $\left(PID_{i},PID_{i}^{new},T_{SK},T_{4},\delta_{new}\right)$ from the normal vehicle $v_{i}$, the fog server $FS_{j}$ firstly checks the newness of the timestamp $T_{4}$ as Equation (5). Additionally, the fog server $FS_{j}$ checks the expiration time of $T_{SK}$.
- The fog server $FS_{j}$ checks the authenticity and validity of signature $\delta_{new}$ as Equation (16).
  (16) $$\begin{array}{cc} \delta_{new}\cdot P & =(SK_{N}+r\cdot h_{2}(PID_{i,1}^{new}||PID_{i,2}^{new}\left|\right|T_{4}))\cdot P\\ \; & =(s\cdot h_{2}(PID_{i,1}||PID_{i,2}\left|\right|T_{SK})+r\cdot h_{2}(PID_{i,1}^{new}||PID_{i,2}^{new}\left|\right|T_{4}))\cdot P\\ \; & =(h_{2}(PID_{i,1}||PID_{i,2}\left|\right|T_{SK})\cdot s\cdot P+h_{2}(PID_{i,1}^{new}||PID_{i,2}^{new}\left|\right|T_{4})r\cdot P)\\ \; & =h_{2}(PID_{i,1}||PID_{i,2}\left|\right|T_{SK})Pub+h_{2}(PID_{i,1}^{new}||PID_{i,2}^{new}\left|\right|T_{4})PID_{i,1} \end{array}$$
- If Equation (16) holds, the fog server $FS_{j}$ prepares a new private key $SK_{N}^{new}$ as Equation (17) with a new life-time ($T_{SK}^{new}$).
  (17) $$SK_{N}^{new}=s\cdot h_{2}(PID_{i,1}^{new}||PID_{i,2}^{new}\left|\right|T_{SK}^{new}))$$
- The fog server $FS_{j}$ encrypts new private key $SK_{N}^{new}$ as $SK_{N-enc}^{new}=SK_{N}^{new}⊕h_{1}\left(s\cdot PID_{i,1}^{new}\right)$ and computes $\delta_{fog}^{new}$ as Equation (18).
  (18) $$\delta_{fog}^{new}=h_{2}(SK_{N-enc}^{new}\left|\right|T_{5}\left|\right|T_{SK}^{new})$$
- The fog server $FS_{j}$ sends $\left(SK_{N-enc}^{new},T_{SK}^{new},T_{5},\delta_{fog}^{new}\right)$ into a normal vehicle $v_{i}$.
- Once receiving the parameters $\left(SK_{N-enc}^{new},T_{SK}^{new},T_{5},\delta_{fog}^{new}\right)$, the vehicle $v_{i}$ decrypts $SK_{N-enc}^{new}$ and checks $\delta_{fog}^{new}$ to accept the new private key with its lifetime.

Note that the normal vehicle in our proposed travels from fog servers to others via different 5G-BS within the VANET system. This means that a vehicle has the ability to renew its pseudonym ID and private key without communicating with TA. As a result, our proposal avoids the single point of failure.

### 4.5. Message Signing Phase

Once vehicle $v_{i}$ wishes to broadcast the message in an open channel environment of 5G-enabled vehicular fog computing, the vehicle must run this phase as follows:

- Vehicle $v_{i}$ generates message $M_{i}$ regarding its road status and current freshness timestamp $T_{i}$.
- Vehicle $v_{i}$ prepares two pseudonym IDs $PID_{i}$ ($PID_{i,1}$ and $PID_{i,2}$) and concerned private key $Sk_{N}$ which was obtained from fog sever.
- Vehicle $v_{i}$ computes a message signature $\delta_{i}$ as Equations (19) and (20) for normal vehicle and COVID-19 vehicle, respectively.
  (19) $$\delta_{i}=Sk_{N}+r\cdot h_{3}(PID_{i,1}||PID_{i,2}\left|\right|M_{i}\left|\right|T_{i})$$
  (20) $$\delta_{i}=Sk_{C}+r\cdot h_{3}(PID_{i,1}||PID_{i,2}\left|\right|M_{i}\left|\right|T_{i})$$
- Vehicle $v_{i}$ then computes $\sigma_{i}=\delta_{i}\cdot P$, which is applied to reduce the number of multiplication operations of ECC. As a result, reducing the overhead of the system from the verifier side in our proposal.
- Normal vehicle $v_{i}$ broadcasts message-tuple $\left(M_{i},PID_{i,1},PID_{i,2},T_{i},T_{SK},\sigma_{i}\right)$ to other normal vehicles or nearby fog servers.

Note that only a normal vehicle executes this phase to sign message and sends it to other vehicles or fog servers, while a COVID-19 vehicle only sends the security parameters with the message to nearby a fog sever through wide-range communication of 5G-BS.

### 4.6. Signature Verification Phase

Once the recipient (fog server or vehicle) receives message-tuple $\left(M_{i},PID_{i,1},PID_{i,2},T_{i},T_{SK},\sigma_{i}\right)$ from vehicle $v_{i}$, the verifier recipients should authenticate and validate the sent message before accepting message $M_{i}$ as follows:

- Checker tests the freshness of timestamp $T_{i}$ of message-tuple $\left(M_{i},PID_{i,1},PID_{i,2},T_{i},T_{SK},\sigma_{i}\right)$ as shown in Equation (5) in order to detect replay attacks.
- Checker uses one of the following processes (single-signature verification or batch signature verification) in order to detect modification, forgery, or MITM attacks.
- Single signature verification process: checker tests whether Equation (21) holds or not.
  (21) $$\begin{array}{c} \sigma_{i}\overset{?}{=}h_{2}(PID_{i,1}||PID_{i,2}\left|\right|T_{SK})\cdot Pub+h_{3}(PID_{i,1}||PID_{i,2}\left|\right|M_{i}\left|\right|T_{i})\cdot PID_{i,1} \end{array}$$
- Batch signature verification process: checker tests whether Equation (22) holds or not.
  (22) $$\begin{array}{c} \sum_{i=1}^{n}\sigma_{i}\overset{?}{=}\sum_{i=1}^{n}h_{2}(PID_{i,1}||PID_{i,2}\left|\right|T_{SK})\cdot Pub+\sum_{i=1}^{n}h_{3}(PID_{i,1}||PID_{i,2}\left|\right|M_{i}\left|\right|T_{i})\cdot PID_{i,1} \end{array}$$

### 4.7. Identity Revocation Phase

In this phase, TA does not only trace the attacker (normal malicious vehicle or malicious COVID-19 vehicle), but also revokes the identity of the malicious vehicle from obtaining the VANET service. This process is as follows:

- Once reporting the forge message-tuple $\left(M_{i},PID_{i,1},PID_{i,2},T_{i},T_{SK},\sigma_{i}\right)$, the fog server computes NVPS for the normal vehicle or CVPS for COVID-19 vehicle as Equation (23) or Equation (24), respectively.
  (23) $$NVPS=PID_{i,2}⊕h_{1}\left(s\cdot PID_{i,1}\right)$$
  (24) $$CVPS=PID_{i,2}⊕h_{1}\left(s\cdot PID_{i,1}\right)$$
- The fog server sends NVPS or CVPS to TA via a secure channel (wired technology).
- TA identify NVPS or CVPS as matched and stored in a normal vehicle registration list and COVID-19 vehicle registration list, respectively, to revoke.
- TA deletes and revokes NVPS or CVPS.
- TA sends the vehicle revocation list into all fog servers.

Note that when the timestamp $T_{SK}$ of private key $SK_{N}$ is close to expiring, the vehicle should request a nearby fog server to update the parameters. In the case that a normal vehicle is revoked, the fog server will discard the process since it was revoked and identified on the vehicle revocation list. At the same time, the fog server will discard the message that was sent from the revoked COVID-19 vehicle before is accepted.

## 5. Security Analysis and Comparison

This section proves the security analysis and comparison of the proposed scheme as follows.

### 5.1. Security Analysis

Our proposed scheme should be satisfying security and privacy requirements in the following steps:

- Authentication and integrity: Before accepting a message, our proposal checks the signature that was attached to a message-tuple $\left(M_{i},PID_{i,1},PID_{i,2},T_{i},T_{SK},\sigma_{i}\right)$. It then only accepts messages that calculate by evaluating Equations (21) and (22). Accordingly, the requirements of a authentication and integrity are applied in our proposal.
- Privacy-preserving: The proposed scheme generates two random numbers *s* and *r* as Pub and $PID_{i,1}=r\cdot P$, respectively. Hence, any attacker attempting to obtain NVPS/CVPS from a message-tuple $\left(M_{i},PID_{i,1},PID_{i,2},T_{i},T_{SK},\sigma_{i}\right)$ will not be capable of doing so without these two numbers. Since $PID_{i,2}=NVPS⊕h_{1}\left(r\cdot Pub\right)$ and $PID_{i,2}=CVPS⊕h_{1}\left(r\cdot Pub\right)$, it becomes a difficult problems. Accordingly, the requirement of privacy-preserving is applied in our proposal.
- Traceability and revocation: Any attacker that attempts to send forged messages or interfere with the operation of the system can be blocked and have their registration revoked by the TA by tracing the message’s source. The vehicle that receives the forged message transmits it to the TA, which performs the steps in Section 4.7. Accordingly, the requirements of traceability and revocation are applied in our proposal.
- Replay attack: Since the timestamp $T_{i}$ is included in a message-tuple $\left(M_{i},PID_{i,1},PID_{i,2},T_{i},T_{SK},\sigma_{i}\right)$, the proposed scheme can avoid replay attacks using Equation (5). Accordingly, a replay attack is resisted in our proposal.
- Forgery attacks: Since the signature tuple is validated by the TA using Equations (21) and (22), no attacker may falsify the identity of a legitimate vehicle sending a message. Accordingly, the forgery attack is resisted in our proposal.
- Modify Attacks: Similar to the forgery attack, it needs an attacker to forge a signature tuple that is validated by computing (21) and (22). This is impossible to fake computationally. Hence, our technique is protected against this attack.
- Man-in-the-middle attack: Because the vehicles communicate directly with one another and are shielded from interference, these types of attacks are not viable.

### 5.2. Security Comparison

This section evaluates and compares the proposed scheme and other related schemes in terms of privacy and security requirements. Table 1 lists the privacy and security comparison. As shown in Table 1, none of these schemes address the COVID-19 virus and solutions for vehicular networks to exchange messages with trusted parts. In order to cope with these issues, this paper proposes efficient mutual authentication that consists of two different aspects according to the special flag (SF) value that involves the mutual authentication process. Based on the SF value, we consider the type of vehicle to be a normal vehicle when SF = 0 and a COVID-19 vehicle when SF = 1.

## 6. Performance Evaluation

### 6.1. Testbed Experiments Using MIRACL

This section presents the test-bed experiments to estimate the running time needed for various cryptographic operations used in the proposed scheme and existing related schemes utilizing the well-known “MIRACL Crypto SDK [33]” which is a C/C++ based library of programming software. For simplicity, the following notations used in this paper are:

- $T_{mul}$ denotes the estimated running time needed for ECC scalar multiplication operation;
- $T_{add}$ denotes the estimated running time needed for the ECC point addition operation P+Q;
- $T_{h}$ denotes the estimated running time needed for the secure cryptographic hash function.

Under this scenario, we have presented the computer setting as follows: “Model: Desktop, Processor: AMD Ryzen 7 5800H with Radeon Graphics, CPU Architecture: 64 bits, OS: Windows 11 Home Single Language with 16 GB memory”. There have been 1000 runs of each primitive. For each primitive, the average times in milliseconds are noted. The experiment’s results based on MIRACL are displayed in Table 2.

### 6.2. Computational Cost and Comparison

In this section, we estimate the computation cost of the operations used in single-message signing, single-message verification, and batch messages verification for our proposed and existing ECC-based related schemes. Table 3 lists a comparison of the computational costs.

The entity in the scheme of Zhang et al. [22] requires two general hash functions and two operations of scalar multiplication for a single-message signing. Accordingly, the whole computation overhead is $2T_{h}+2T_{mul}\approx 2*0.0001+2*0.6718\approx 1.3456$ ms, while an entity needs two operations of point additions, two general hash functions, and three operations of scalar multiplication for single-message verification. Accordingly, the whole computation overhead is $2T_{add}+2T_{h}+3T_{mul}\approx 2*0.0031+2*0.0001+3*0.6718\approx 2.6923$ ms. Additionally, the entity needs (*n*) operations of point addition, 2*n* general hash functions, and (2*n* + 2) operations of scalar multiplication for batch message verification. Accordingly, the whole computation overhead is $nT_{add}+2nT_{h}+\left(2n+2\right)T_{mul}\approx 1.3436+1.3487n$ ms.

The entity in the scheme of Li et al. [23] requires two general hash functions and one operation of scalar multiplication for single-message signing. Accordingly, the whole computation overhead is $2T_{h}+2T_{mul}\approx 2*0.0001+1*0.6718\approx 0.6729$ ms, while the entity needs one operation of point additions, two general hash functions, and four operations of scalar multiplication for single-message verification. Accordingly, the whole computation overhead is $1T_{add}+2T_{h}+4T_{mul}\approx 1*0.0031+2*0.0001+3*0.6718\approx 2.0236$ ms. Additionally, the entity needs (*n*) operations of point addition, 2*n* general hash functions, and (*n* + 2) operations of scalar multiplication for batch message verification. Accordingly, the whole computation overhead is $nT_{add}+2nT_{h}+\left(n+2\right)T_{mul}\approx 1.3436+1.3487nn$ ms.

The entity in the scheme of Cui et al. [24] requires three general hash functions and three operations of scalar multiplication for a single-message signing. Accordingly, the whole computation overhead is $3T_{h}+3T_{mul}\approx 3*0.0001+3*0.6718\approx 2.0157$ ms, while the entity needs one operation of point addition, two general hash functions, and three operations of scalar multiplication for single-message verification. Accordingly, the whole computation overhead is $1T_{add}+2T_{h}+4T_{mul}\approx 0.0031+2*0.0001+3*0.6718\approx 2.0187$ ms. Additionally, the entity needs (*n* − 1) operations of point addition, 2*n* general hash functions, and (*n* + 2) operations of scalar multiplication for batch message verification. Accordingly, the whole computation overhead is $n-1T_{add}+2nT_{h}+\left(n+2\right)T_{mul}\approx 1.3405+0.6782n$ ms.

The entity in the scheme of Alshudukhi et al. [25] requires two general hash functions and two operations of scalar multiplication for single-message signing. Accordingly, the whole computation overhead is $2T_{h}+2T_{mul}\approx 2*0.0001+2*0.6718\approx 1.3438$ ms, while the entity needs one operation of point addition, two general hash functions, and three operations of scalar multiplication for single-message verification. Accordingly, the whole computation overhead is $1T_{add}+2T_{h}+4T_{mul}\approx 0.0031+2*0.0001+3*0.6718\approx 2.0187$ ms. Additionally, the entity needs (*n*) operations of point addition, 2*n* general hash functions, and (*n* + 2) operations of scalar multiplication for the batch message verification. Accordingly, the whole computation overhead is $nT_{add}+2nT_{h}+\left(n+2\right)T_{mul}\approx 1.3436+0.6782n$ ms.

The entity in the scheme of Al-Shareeda et al. [26] requires two general hash functions and one operation of scalar multiplication for a single-message signing. Accordingly, the whole computation overhead is $2T_{h}+1T_{mul}\approx 2*0.0001+0.6718\approx 0.672$ ms, while the entity needs one operation of point addition, two general hash functions, and four operations of scalar multiplication for single-message verification. Accordingly, the whole computation overhead is $1T_{add}+2T_{h}+4T_{mul}\approx 0.0031+2*0.0001+4*0.6718\approx 2.6905$ ms. Additionally, the entity needs (*n*) operations of point addition, 2*n* general hash functions, and (2*n* + 2) operations of scalar multiplication for batch message verification. Accordingly, the whole computation overhead is $nT_{add}+2nT_{h}+\left(2n+2\right)T_{mul}\approx 1.3436+1.3469n$ ms.

The entity in our proposed scheme requires one operation of point addition, one general hash function, and one operation of scalar multiplication for a single-message signing. Accordingly, the whole computation overhead is $1T_{add}+1T_{h}+1T_{mul}\approx 0.0031+0.0001+0.6718\approx 0.675$ ms, while the entity needs one operation of point addition, two general hash functions, and two operations of scalar multiplication for single-message verification. Accordingly, the whole computation overhead is $1T_{add}+2T_{h}+2T_{mul}\approx 0.0031+2*0.0001+2*0.6718\approx 1.3469$ ms. Additionally, the entity needs (*n* + 1) operations of point addition, *n* general hash functions, and two operations of scalar multiplication for batch message verification. Accordingly, the whole computation overhead is $\left(n+1\right)T_{add}+nT_{h}+2T_{mul}\approx 1.3467+0.0032n$ ms.

### 6.3. Communication Cost and Comparison

In this section, we estimate the communication costs of the item size used as the final exchanged message among entities for our proposed and existing ECC-based related schemes. The major concentration is the communication overhead consisting of the pseudonym IDs, signatures, and timestamps for the message-signature tuples. Referring to [13,17,34], we suppose that the bit lengths for the timestamp, hash function, element in $Z_{q}^{*}$, and element in *G* are 32, 160, 160, 320 bits, respectively. Table 4 lists a comparison of communication costs of the proposed scheme and related works.

In scheme of Zhang et al. [22], the entity broadcasts a message-tuple $\left\{ID_{i,1},ID_{i,2},M,T,\sigma \right\}$ to other vehicles, where 320×2+32+160≈832. In the scheme of Li et al. [23], the entity broadcasts a message-tuple $\left\{M_{i},PID_{i,l},PK_{i,l},R_{i},T_{i},sig_{i}\right\}$ to other vehicles, where 320×3+160+32≈1152. In the scheme of Cui et al. [24], the entity broadcasts a message-tuple $\left\{DT_{ij},D_{j},PID_{j},\delta_{j},T_{j}\right\}$ to other vehicles, where 32+2×160+2×320≈992 bits. In the scheme of Alshudukhi et al. [25], the entity broadcasts a $\left\{PsID_{i}^{1},PsID_{i}^{2},m_{i},TS_{i},\sigma_{m_{i}}\right\}$ to other vehicles, where 32+2×160+1×320≈672 bits. In the scheme of Al-Shareeda et al. [26], the entity broadcasts a message-tuple $\left\{M_{i},PK_{i,l},PID_{i,l},D_{i},T_{i},\delta_{i}\right\}$ to other vehicles, where 2×320+2×160+32≈992 bits. In our proposal, the entity broadcasts a message-tuple $\left(M_{i},PID_{i,1},PID_{i,2},T_{i},T_{SK},\sigma_{i}\right)$ to other vehicles, where 2×32+160+2×320≈864 bits.

Hence, according to the above analysis, this paper proves that the communication costs of each element (as can be seen in Section 4.5) in message-tuple is lower compared with recent studies.

### 6.4. Simulation Environment

This section implements the proposed scheme in a simulation environment in order to evaluate the performance.

**Figure 4:**
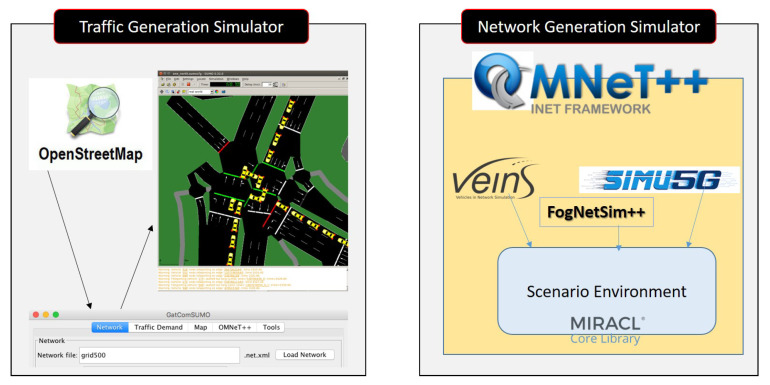
Simulation Environment of Proposed Scheme.

As listed in Figure 4, the proposed scheme generates the traffic simulator and network simulator using SUMO [35] and OMNeT++ [36], receptively. Additionally, the proposed scheme applies tools and frameworks such as OpenStreetMap [37], GatcomSUMO [38], VEINS [39], FogNetSim++ [40], Simu5G [41], and MIRACL [33] to implement a simulation environment in an urban area for 5G-enabled vehicular fog computing. The simulation environment’s settings are listed in Table 5.

**Figure 5:**
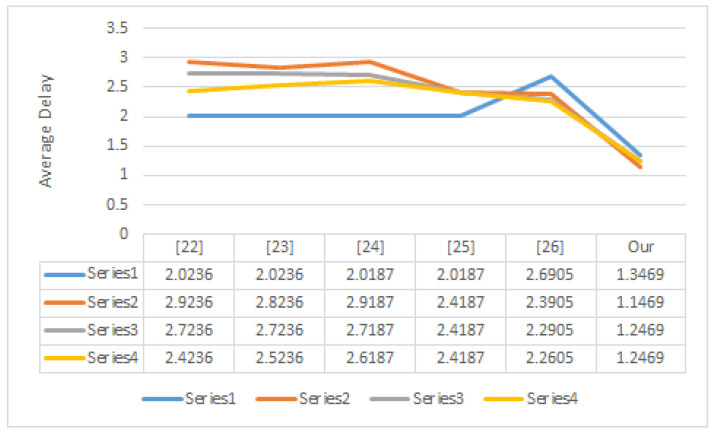
Average Delay for Verifying a Single Message.

Each cryptographic operation has its own distinct runtime, and that is what is used to calculate the total time. The overhead cost is the amount of time that has passed since the exit and the entrance (see Equation (25)).

(25) $$ET=\frac{1}{M}\sum_{i=1}^{n}M\left(T_{out}^{i}-T_{in}^{i}\right)$$

where $T_{in}^{i}$ is the message entrance time *i*, *M* is the message number, and $T_{out}^{i}$ is the message exit time *i*. Figure 5 depicts the average time to verify a single message between the proposed and existing schemes.

## 7. Conclusions

In this paper, the development of an efficient mutual authentication scheme for healthcare solutions in 5G-enabled vehicular fog computing places emphasis on controlling automotive pandemics in intelligent transportation systems. In the proposed scheme, there is no requirement to broadcast messages (e.g., velocity, location, speed, direction) to others for COVID-19 vehicles. Thus, the COVID-19 vehicle only sends a message (e.g., healthcare information) to the nearest fog server through the communication range of 5G-BS using its private key. Security analysis shows that the proposed scheme satisfies privacy and security requirements as well as achieves COVID-19 and healthcare solutions. The performance section evaluates whether the proposed scheme is more efficient in terms of communication and computation costs as compared to the most recent related works.

## Figures and Tables

**Table 1:** Comparison of Privacy and Security Requirements.

Requirements	[22]	[23]	[24]	[25]	[26]	Our
Privacy-preserving	✔	✔	✔	✔	✔	✔
Authentication	✔	✔	✔	✔	✔	✔
Integrity	✔	✔	✔	✔	✔	✔
Security attacks resistance	✔	✔	✔	✔	✔	✔
Traceability	✔	✔	✔	✔	✔	✔
Revocability	✘	✘	✘	✔	✘	✔
Not RSU-based	✔	✘	✔	✘	✔	✔
COVID-19 solution	✘	✘	✘	✘	✘	✔
Healthcare solutions	✘	✘	✘	✘	✘	✔

**Table 2:** Notation with its Costs of Running Time.

Notation	Running Time
$T_{mul}$	0.6718 ms
$T_{add}$	0.0031 ms
$T_{h}$	0.0001 ms

**Table 3:** The Cost of Computation of Authentication Schemes.

Scheme	MsgSign Phase	SigVerify Phase	BSigVerify Phase
[22]	$2T_{h}+2T_{mul}\approx 2*0.0001+2*0.6718\approx 1.3456$ ms	$2T_{add}+2T_{h}+3T_{mul}\approx 2*0.0031+2*0.0001+3*0.6718\approx 2.0236$ ms	$nT_{add}+2nT_{h}+\left(n+2\right)T_{mul}\approx 1.3436+1.3487nn$ ms
[23]	$2T_{h}+2T_{mul}\approx 2*0.0001+1*0.6718\approx 0.6729$ ms	$1T_{add}+2T_{h}+4T_{mul}\approx 1*0.0031+2*0.0001+3*0.6718\approx 2.0236$ ms	$nT_{add}+2nT_{h}+\left(2n+2\right)T_{mul}\approx 1.3436+1.3487n$ ms
[24]	$3T_{h}+3T_{mul}\approx 3*0.0001+3*0.6718\approx 2.0157$ ms	$1T_{add}+2T_{h}+4T_{mul}\approx 0.0031+2*0.0001+3*0.6718\approx 2.0187$ ms	$n-1T_{add}+2nT_{h}+\left(n+2\right)T_{mul}\approx 1.3405+0.6782n$ ms
[25]	$2T_{h}+2T_{mul}\approx 2*0.0001+2*0.6718\approx 1.3438$ ms	$1T_{add}+2T_{h}+4T_{mul}\approx 0.0031+2*0.0001+3*0.6718\approx 2.0187$ ms	$nT_{add}+2nT_{h}+\left(n+2\right)T_{mul}\approx 1.3436+0.6782n$ ms
[26]	$2T_{h}+1T_{mul}\approx 2*0.0001+0.6718\approx 0.672$ ms	$1T_{add}+2T_{h}+4T_{mul}\approx 0.0031+2*0.0001+4*0.6718\approx 2.6905$ ms	$nT_{add}+2nT_{h}+\left(2n+2\right)T_{mul}\approx 1.3436+1.3469n$ ms
Our	$1T_{add}+1T_{h}+1T_{mul}\approx 0.0031+0.0001+0.6718\approx 0.675$ ms	$1T_{add}+2T_{h}+2T_{mul}\approx 0.0031+2*0.0001+2*0.6718\approx 1.3469$ ms	$\left(n+1\right)T_{add}+nT_{h}+2T_{mul}\approx 1.3467+0.0032n$ ms

**Table 4:** Comparison of Communication Costs.

Scheme	Message-Signature Tuple	Size (bits)	*n* Size (bits)
[22]	$\left\{ID_{i,1},ID_{i,2},M,T,\sigma \right\}$	320×2+32+160≈832	832 *n*
[23]	$\left\{M_{i},PID_{i,l},PK_{i,l},R_{i},T_{i},sig_{i}\right\}$	320×3+160+32≈1152	1152 *n*
[24]	$\left\{DT_{ij},D_{j},PID_{j},\delta_{j},T_{j}\right\}$	32+2×160+2×320≈992	992 *n*
[25]	$\left\{PsID_{i}^{1},PsID_{i}^{2},m_{i},TS_{i},\sigma_{m_{i}}\right\}$	2×320+2×160+32≈992	992 *n*
[26]	$\left\{M_{i},PK_{i,l},PID_{i,l},D_{i},T_{i},\delta_{i}\right\}$	2×320+2×160+32≈992	992 *n*
Our	$\left(M_{i},PID_{i,1},PID_{i,2},T_{i},T_{SK},\sigma_{i}\right)$	2×32+160+2×320≈864	864 *n*

**Table 5:** Simulation Environment Parameters.

Parameter	Value
Playground size	x = 3463 m, y = 4270 m and z = 50 m
Maximum transmission	20 mW
Simulation time	200 s
Bit rate	6 Mbps
Physical layer	IEEE 802.11p
Mac layer	IEEE 1609.4

## Data Availability

Not Applicable.
